# Dosimetric Effects of the Interfraction Variations during Whole Breast Radiotherapy: A Prospective Study

**DOI:** 10.3389/fonc.2015.00199

**Published:** 2015-09-16

**Authors:** Julian Jacob, Steve Heymann, Isabelle Borget, Isabelle Dumas, Elyes Riahi, Pierre Maroun, Patrick Ezra, Elena Roberti, Sofia Rivera, Eric Deutsch, Céline Bourgier

**Affiliations:** ^1^Department of Radiotherapy, Gustave Roussy, Villejuif, France; ^2^Department of Biostatistics and Epidemiology, Gustave Roussy, Villejuif, France; ^3^University Paris-Sud, Kremlin-Bicêtre, France; ^4^Department of Physics, Gustave Roussy, Villejuif, France; ^5^Department of Oncologic Radiotherapy, Institut du Cancer de Montpellier, Montpellier, France

**Keywords:** dosimetry, interfraction variations, breast radiotherapy, central lung distance, inferior central margin

## Abstract

**Introduction:**

The aim of this work was to assess the dosimetric impact of the interfraction variations during breast radiotherapy.

**Materials and methods:**

Daily portal imaging measurements were prospectively performed in 10 patients treated with adjuvant whole breast irradiation (50 Gy/25 fractions). Margins between the clinical target volume and the planning target volume (PTV) were 5 mm in the three dimensions. Parameters of interest were the central lung distance (CLD) and the inferior central margin (ICM). Daily movements were applied to the baseline treatment planning (TP1) to design a further TP (TP2). The PTV coverage and organ at risk exposure were measured on both TP1 and TP2, before being compared.

**Results:**

A total of 241 portal images were analyzed. The random and systematic errors were 2.6 and 3.7 mm for the CLD, 4.3 and 6.9 mm for the ICM, respectively. No significant consequence on the PTV treatments was observed (mean variations: +0.1%, *p* = 0.56 and −1.8%, *p* = 0.08 for the breast and the tumor bed, respectively). The ipsilateral lung and heart exposure was not significantly modified.

**Conclusion:**

In our series, the daily interfraction variations had no significant effect on the PTV coverage or healthy tissue exposure during breast radiotherapy.

## Introduction

Breast-conserving surgery and adjuvant radiotherapy are the standard treatments of patients with early-stage breast cancer (BC). Postoperative irradiation is pivotal as it lessens the relative risk of local failure (absolute reduction of 15.7% at 10 years) and, therefore, the probability of BC death (absolute reduction of 3.8% at 15 years) (1). During the last decades, the modalities of breast radiotherapy evolved from two-dimensional irradiation to intensity-modulated radiation therapy (IMRT) (2). Three-dimensional (3D) conformal radiotherapy is the technique most commonly used in BC treatment. To do so, the target volumes (gross tumor volume, GTV; clinical target volume, CTV) and healthy organs are delineated on a computed tomography (CT) scan. Then, margins are added to define the planning target volume (PTV) considering the internal organ motion and the patient setup error (3). Portal imaging (PI) plays a key role in the patient positioning management and, consequently, in the treatment quality. Target volume motion can be studied using two parameters: the interfraction movements, measured between two different treatment days, and the intrafraction variations, assessed during one single radiotherapy session (4).

Although the patient setup uncertainties are considered in the PTV delineation, interfraction movements should be limited in order to reduce the risk of inadequate target volume coverage and/or to prevent the occurrence of toxicities. This work had for purpose to assess the setup variations during whole breast radiotherapy using daily PI, a simple and reproducible technique (5–7). The relevance of the PTV margins was evaluated in order to improve the treatment quality.

We prospectively studied the role of the interfraction variations on the target volume coverage and healthy tissue exposure during radiotherapy indicated for early-stage BC. Intrafraction movements were not considered in this work.

## Materials and Methods

This study was carried out in accordance with the recommendations edited by the Head Committee of the Gustave Roussy Radiotherapy Department with written informed consent from all subjects. All patients gave written informed consent in accordance with the Declaration of Helsinki.

### Population description

A cohort of 10 consecutive patients (mean age: 57.5 years; range: 39–77) was treated for early-stage BC with breast-conserving surgery and axillary dissection or sentinel node biopsy followed by adjuvant whole breast irradiation. A left-sided tumor was reported in six patients and a right-sided one in four patients.

All patients had whole breast irradiation (50 Gy in 25 fractions over 5 weeks) followed by a tumor bed boost (16 Gy in 8 fractions over 2 weeks). Regional lymph node irradiation (supra- and infraclavicular, axillary, internal mammary nodes) was not considered in this study.

### Simulation and treatment planning

The simulation was completed using a CT scan without iodine contrast agent infusion (Siemens Somatom^®^ Sensation Open – 24 slices). Patients were in the supine position on the Med Tec (Model MT-350-N) inclined breast board with both their arms up and behind the head. Image acquisition was performed from the neck to the upper abdomen in 3-mm slices using the free-breathing technique. No immobilization device was used. The clinical mammary gland borders, lumpectomy scar, and post-surgical induration were outlined with radio-opaque wires.

The GTV had been removed and was not delineated. The clinical target volume 1 (CTV1) was defined by the mammary gland delineation: (i) anterior border, 5 mm under the skin; (ii) posterior border, the upper face of the pectoralis major muscle and the ribs; (iii) medial border, the clinical and radiological root; (iv) lateral border, the clinical and radiological marker; (v) superior border, the upper clinical and radiological limit encompassing the axillary extension of the mammary gland; (vi) inferior border, the infra-mammary fold and/or the lower clinical marker in case of large breast size. The clinical target volume 2 (CTV2) corresponded to the tumor bed, identified by the breast remodeling and the surgical clips, with a 15-mm expansion in three dimensions (8, 9). Furthermore, a 5-mm margin was added to the CTV1 and CTV2 in three dimensions to create the planning target volumes 1 and 2 (PTV1 and PTV2, respectively). The following healthy organs were delineated: the ipsilateral lung and the heart (from the base till the level of the bifurcation of the pulmonary artery).

Dosimetric optimization was performed in the transverse, sagittal, and coronal planes (treatment planning 1: TP1) using the Dosisoft^®^/Isogray^®^ (v4.1) treatment planning system. The patients were treated with 6-MV X-ray tangential beams. Wedges were used to improve the PTV coverage and dose homogeneity, which should be kept between 95 and 107% of the prescribed dose (3). Doses and volumes were systematically recorded.

### Patient positioning control and study parameters: CLD and ICM

**Figure 1:**
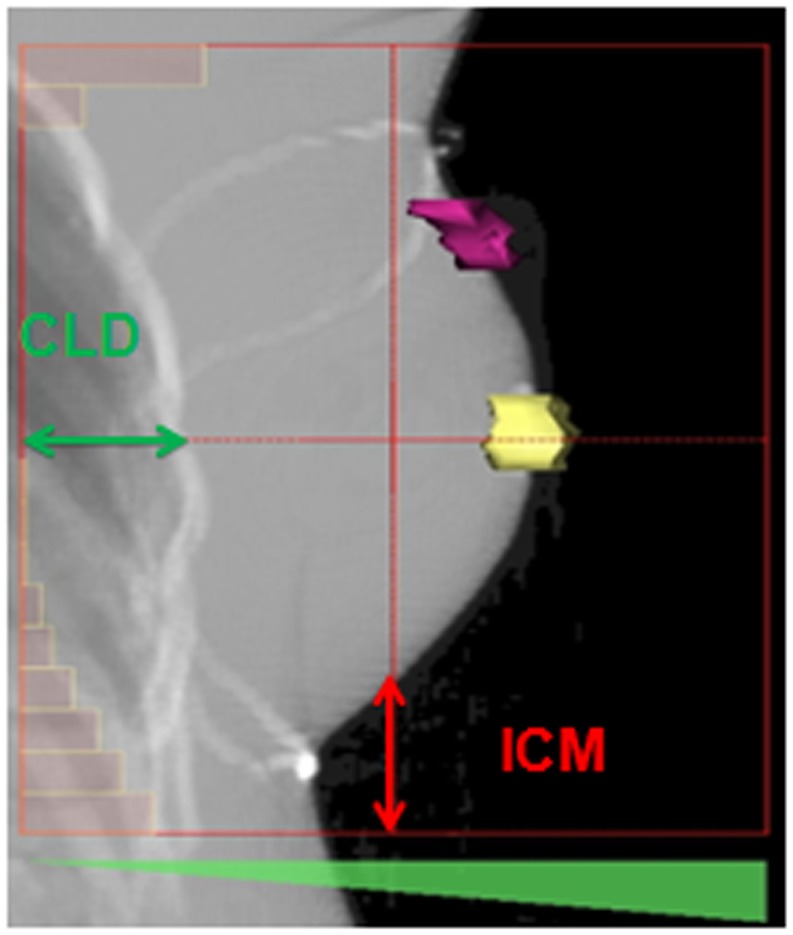
**Description of the inferior central margin and central lung distance on a digital reconstructed radiography performed on the treatment planning system Isogray^®^ according to Fein et al. (6) and Kong et al. (10)**. The nipple and the surgical scar are, respectively, delineated in yellow and purple. CLD, central lung distance; ICM, inferior central margin.

Interfraction movements were daily assessed using an electronic PI device. The portal images of the medial tangential beam treating the whole mammary gland were acquired before each of the 25 first fractions. The central lung distance (CLD) and the inferior central margin (ICM) (Figure 1) were measured on the first acquired portal image by the medical physicist and compared with the digital reconstructed radiography (DRR) (6, 10). The CLD and ICM were defined to assess the interfraction variations along the anteroposterior and craniocaudal axes, respectively. The CLD and ICM were delineated on every daily portal image by the medical physicist. The measured data were then verified by both the medical physicist and the radiation oncologist. According to the methods reported in previously published articles, the measurements were performed on portal images (Figure 1), so that the lateral shifts could not be quantified (6, 7, 11, 12).

The DRR, created from the simulation data, was the reference image for every radiotherapy session. The interfraction variations evaluation was based on the comparison of the CLD and ICM estimated on the daily portal images with the DRR data.

Considering the CLD and ICM individually, the SD, random, and systematic errors were assessed. The SD was determined according to the formula reported by Fein et al. (6). The random and systematic errors were estimated using the methods published by Rosenthal et al. (13). The random error corresponds to the deviation of the data measured on the daily portal images from the mean values. The systematic error is the difference between the mean values and the simulation data (13).

### Effects of the interfraction variations on the target volume coverage and OAR exposure

For each patient, an additional treatment planning was performed considering the CLD and ICM mean values (treatment planning 2: TP2). TP2 was compared with the baseline treatment planning (TP1) to assess the role of the daily interfraction variations on the target volume coverage and organ at risk (OAR) exposure. The following dosimetric parameters were studied: the volumes encompassing 95% of PTV1 (PTV1 V95) and 95% of PTV2 (PTV2 V95), for the ipsilateral lung; the percentages of volume receiving 20 and 30 Gy (V20 and V30, respectively), for the heart; V30 and the mean dose. The differences in terms of PTV coverage and OAR exposure were expressed in relative and absolute values, respectively.

### Statistical analyses

Quantitative data were expressed in mean values and SDs considering the sample size. Qualitative data were expressed in percentages. TP2 results were compared with those obtained from TP1 using a Wilcoxon test for the mean values as the variables were non-normally distributed, whereas qualitative data were compared using a chi-square test. Significance level was 0.05. Statistics were performed using the Statistical Analysis System software SAS^®^ 9.2 (SAS Institute Incorporation, Cary, NC, USA).

## Results

### Initial treatment planning

On the DRR, the mean CLD was 20.8 mm (range 16.3–26.4 mm) and the mean ICM 27.1 mm (range 13.1–39.0 mm) (Table 1). The CLD exceeded 20 mm in 116 portal images (48.7% of the total number assessed) and 25 mm in 43 (18.1%). For one patient, due to the superficial isocenter position, the ICM could not be measured (patient #9).

**Table 1:** **Individual dosimetric data according to the baseline simulation (treatment planning 1)**.

Patient	CLD (mm)	ICM (mm)	PTV1 V95 (cc)	PTV2 V95 (cc)	Ipsilateral lung V20 (%)	Ipsilateral lung V30 (%)	Heart V30 (%)	Mean heart dose (Gy)
1	16.3	39.0	214.1	54.4	6.7	6.1	0	0.9
2	17.6	13.1	1255.4	481.9	8.3	7.4	NA	NA
3	19.0	32.6	500.5	191.1	8.9	7.7	4.0	4.7
4	17.5	34.7	1868.6	448	9.1	8.0	NA	NA
5	18.6	13.2	1376.8	426.4	9.2	8.5	NA	NA
6	26.4	26.9	829.1	277.6	12.2	10.8	0.2	1.7
7	20.0	33.4	1017.2	370.8	11.2	10.0	1.2	2.6
8	22.9	19.7	1392.9	414.1	10.2	9.4	NA	NA
9	24.0	NA	573.0	213.4	14.7	11.3	1.4	2.3
10	25.3	31.0	1072.5	228.3	16.2	14.5	2.6	3.5

*CLD, central lung distance; ICM, inferior central margin; NA, not assessable; PTV1, planning target volume 1 (whole mammary gland); PTV2, planning target volume 2 (tumor bed); TP1, treatment planning 1; TP2, treatment planning 2; V20, volume receiving at least 20 Gy; V30, volume receiving at least 30 Gy; V95, volume receiving at least 95% of the prescribed dose*.

The mean CTV1 was 559.2 cc (range 35.6–951.7 cc) and the mean PTV1 719.8 cc (range 90.7–1182.9 cc). The mean CTV2 was 60.6 cc (range 33.6–92.2 cc) and the mean PTV2 93.9 cc (range 11.4–148.8 cc).

The mean ipsilateral lung dose was 6.8 Gy (range 4.5–9.3 Gy). The mean ipsilateral lung V20 and V30 were 10.7% (range 6.7–16.2%) and 9.4% (range 6.1–14.5%), respectively (Table 1). In case of left-sided BC (*n* = 6), the mean heart dose was 2.6 Gy (range 0.9–4.7 Gy) and the mean heart V30 1.6% (range 0–4.0%) (Table 1).

### Interfraction variations

A total of 241 portal images were analyzed (Table 2). Considering the whole cohort, the mean CLD measured on portal images was 19.9 mm (range 11.7–26.3 mm) and the mean ICM was 22.8 mm (range 9.0–34.0 mm) (Table 2).

**Table 2:** **Individual dosimetric data considering the mean interfraction variations (treatment planning 2)**.

Patient	Number of acquired portal images	CLD (mm)	ICM (mm)	PTV1 V95 (cc)	PTV2 V95 (cc)	Ipsilateral lung V20 (%)	Ipsilateral lung V30 (%)	Heart V30 (%)	Mean heart dose (Gy)
1	25	16.7	34.0	260.8	61.2	8.0	7.4	0	0.8
2	25	14.4	12.9	1186.4	465.2	6.5	5.8	NA	NA
3	25	19.5	21.4	527.1	179.1	10.8	9.4	4.1	3.9
4	25	11.7	34.1	1570.5	391.2	4.5	3.4	NA	NA
5	20	22.5	9.0	1456.5	429.9	11.1	9.8	NA	NA
6	24	26.3	19.8	818.1	268.4	12.5	11.2	0.2	1.5
7	25	19.5	29.0	1039.6	373.9	11.6	10.3	1.3	2.4
8	22	19.4	18.1	1256.3	395	7.6	6.8	NA	NA
9	25	25.2	NA	610.5	217	14.6	13.2	2.1	2.6
10	25	24.4	27.1	986.2	220.7	14.2	12.5	1.7	2.8

*CLD, central lung distance; ICM, inferior central margin; NA, not assessable; PTV1, planning target volume 1 (whole mammary gland); PTV2, planning target volume 2 (tumor bed); TP1, treatment planning 1; TP2, treatment planning 2; V20, volume receiving at least 20 Gy; V30, volume receiving at least 30 Gy; V95, volume receiving at least 95% of the prescribed dose*.

One SD was estimated at 5.3 and 9.4 mm for the CLD and the ICM, respectively. The random and systematic errors were 2.6 and 3.7 mm for the CLD, 4.3 and 6.9 mm for the ICM, respectively.

### Effects of the interfraction variations on the PTV coverage and OAR exposure

The interfraction movements did not significantly affect the PTV1 V95 and PTV2 V95 coverage (respective mean variations estimated at +0.1%, *p* = 0.56; and −1.8%, *p* = 0.08). However, a mean extension of 8.3% (range 2.2–21.8%) was observed for PTV1 V95 in five patients. The PTV2 V95 was increased in four patients (mean value: 3.9%, range 0.8–12.5%) (Tables 2 and 3). On the other hand, the PTV1 V95 and PTV2 V95 were lessened in five and six patients with mean decreases of 8.1% (range 1.3–15.9%) and 5.6% (range 3.3–12.7%), respectively.

**Table 3:** **Planning target volume coverage variations**.

Patient	PTV1 V95 (cc)		Variation (%)	PTV2 V95 (cc)		Variation (%)
	TP1	TP2		TP1	TP2	
1	214.1	260.8	+21.8	54.4	61.2	+12.5
2	1255.4	1186.4	−5.5	481.9	465.2	−3.5
3	500.5	527.1	+5.3	191.1	179.1	−6.3
4	1868.6	1570.5	−15.9	448	391.2	−12.7
5	1376.8	1456.5	+5.8	426.4	429.9	+0.8
6	829.1	818.1	−1.3	277.6	268.4	−3.3
7	1017.2	1039.6	+2.2	370.8	373.9	+0.8
8	1392.9	1256.3	−9.8	414.1	395	−4.6
9	573.0	610.5	+6.5	213.4	217	+1.7
10	1072.5	986.2	−8.0	228.3	220.7	−3.3

*PTV1, planning target volume 1 (whole mammary gland); PTV2, planning target volume 2 (tumor bed); TP1, treatment planning 1; TP2, treatment planning 2; V95, volume receiving at least 95% of the prescribed dose*.

When the interfractions movements were applied to TP1, the mean heart dose variation was 0.3 Gy (*p* = 0.15) (Tables 2 and 4). The mean differences in terms of ipsilateral lung V20, V30, and heart V30 were, respectively, 0.5 (*p* = 0.45), 0.4 (*p* = 0.59), and 0 points (*p* = 1). For all patients, whatever the treatment planning considered, the ipsilateral lung V20, V30, and the heart V30 did not exceed 20, 15, and 5%, respectively.

**Table 4:** **Organ at risk exposure variations**.

Patient	Ipsilateral lung V20 (%)		Variation (points)	Ipsilateral lung V30 (%)		Variation (points)	Heart V30 (%)		Variation (points)	Mean heart dose (Gy)		Variation (Gy)
	TP1	TP2		TP1	TP2		TP1	TP2		TP1	TP2	
1	6.7	8.0	+1.3	6.1	7.4	+1.3	0	0	0	0.9	0.8	−0.1
2	8.3	6.5	−1.8	7.4	5.8	−1.6	NA	NA	NA	NA	NA	NA
3	8.9	10.8	+1.9	7.7	9.4	+1.7	4.0	4.1	+0.1	4.7	3.9	−0.8
4	9.1	4.5	−4.6	8.0	3.4	−4.6	NA	NA	NA	NA	NA	NA
5	9.2	11.1	+1.9	8.5	9.8	+1.3	NA	NA	NA	NA	NA	NA
6	12.2	12.5	+0.3	10.8	11.2	+0.4	0.2	0.2	0	1.7	1.5	−0.2
7	11.2	11.6	+0.4	10.0	10.3	+0.3	1.2	1.3	+0.1	2.6	2.4	−0.2
8	10.2	7.6	−2.6	9.4	6.8	−2.6	NA	NA	NA	NA	NA	NA
9	14.7	14.6	−0.1	11.3	13.2	+1.9	1.4	2.1	+0.7	2.3	2.6	+0.3
10	16.2	14.2	−2.0	14.5	12.5	−2.0	2.6	1.7	−0.9	3.5	2.8	−0.7

*V20, volume receiving at least 20 Gy; V30, volume receiving at least 30 Gy; TP1, treatment planning 1; TP2, treatment planning 2; NA, not assessable*.

## Discussion

The main purpose of this prospective study was to prospectively assess the interfraction variations in patients treated with 3D conformal breast radiotherapy. In our series, the patient setup errors did not significantly affect either the PTV coverage or the healthy tissue exposure.

Using a technique close to ours, Smith et al. studied the interfraction variations by measuring the CLD on portal images in eight patients managed for BC (7). The treatment simulation was performed using a fluoroscopic imaging device or a CT scan. The mean CLD was 1.33 cm (0.59–2.94), and patients repositioning implied CLD variations ranging from 0.38 to 1.62 cm. Moreover, this study showed a correlation between the CLD and the OAR exposure (ipsilateral lung, heart).

Other devices can be used to estimate the patient setup. Baroni et al. also assessed the interfraction movements on four patients referred for left breast radiotherapy (14). Measurements were performed using an opto-electronic position detection system. After considering the breathing movements, the mean 3D shifts reported for the different markers (sternal tattoo points, upper and lower field limits) were reduced to almost 2 mm. The dosimetric effects of these setup errors were limited: the mean variations of the CTV receiving <95% and more than 107% of the daily dose were 1.0 and 0.4%, respectively. Similar trends were reported regarding the ipsilateral lung and heart exposure. The dosimetric consequences of the interfraction variations were not statistically significant. These results tend to show the interest of the opto-electronic localization device to optimize the patient positioning.

If the tattoo points and field borders remain commonly used to set up the patients, the isocenter position has also to be considered. According to Prabhakar et al., the doses administered to the heart, both lungs and contralateral breast significantly increased due to an isocenter shift, particularly in the posterior direction (15). The authors suggested a setup error in isocenter lower to 3 mm to avoid potential complications induced by radiotherapy.

In a prospective series of 10 patients treated in the prone position, Mitchell et al. assessed the inter- and intrafraction variations using an electronic PI device (12). The measurements were performed on the portal images of the medial tangential beam treating the whole breast. The authors took into account the gantry angle deviation from the horizontal axis in order to evaluate more accurately the patient setup along the anteroposterior axis. This method was used due to the respiratory motion variability in the sagittal plane. In this study, the median correction related to the gantry angle was estimated at 2% and considered as negligible. Therefore, in our work, we did not calculate this parameter.

In our study, the random and systematic errors remained below 5 mm for the CLD. Regarding the ICM, the random and systematic errors were assessed at 4.3 and 6.9 mm, respectively. As the target volume coverage and OAR exposure were not significantly modified by the interfraction variations, 5-mm margins seem relevant to delineate the PTV. Nevertheless, a 7-mm expansion along the craniocaudal axis can be discussed.

If the presented results are close to those reported in the literature (16–23), they have to be cautiously interpreted. The CLD and ICM are measured using a two-dimensional imaging, whereas breast radiotherapy is performed using the 3D conformal technique. The target volume deformation, rotation, and shrinkage are not considered in the portal image analysis (24). The lateral shifts have not been assessed in our work. The cone beam-CT (CBCT) provides 3D anatomical information and can reduce the patient positioning uncertainties. The random and systematic errors decreased using CBCT imaging (25). Similar results were observed with cone beam tomosynthesis (26, 27).

In our study, the consequences of the interfraction movements on the PTV coverage have been assessed by the V95 individual variations. Meanwhile, other parameters, such as the conformity index or the homogeneity index, have been used to evaluate the target volume treatment in 3D conformal radiotherapy (28–30). In this small cohort (10 patients), no relevant subgroup analysis could be performed.

Moreover, the intrafraction movements, especially the respiratory motion, have not been measured in our work. During one-single session, especially in IMRT, breathing movements can lead not only to PTV dose heterogeneity but also to increased lung and heart doses (31). Qi et al. reported the dosimetric variations during one breathing cycle in patients treated with 3D conformal radiotherapy for early-stage BC (32). Modifications of the tumor bed coverage were estimated between 1 and 5% but the most significant consequences were described for the internal mammary node treatment: up to 28% of the volume receiving a minimum dose of 45 Gy. No significant difference was reported regarding the healthy tissue exposure. The dosimetric effects of the breathing movements and the isocenter shifts were assessed in 16 patients by Furuya et al. using three radiotherapy techniques: conventional wedge, field-in-field, and irregular surface compensator plan (33). The impacts of respiratory motion were similar from one technique to another. The published results showed a significant effect of the isocenter position discrepancies along the anteroposterior axis: reduction of the CTV V95 and increase of the ipsilateral lung V20. The most important dosimetric consequences were reported with the irregular surface compensator technique.

The effects of the setup uncertainties were also described in patients treated with IMRT for BC (34–36). Considering the wedge, simple, and full IMRT radiation techniques, van Mourik et al. estimated random and systematic errors at about 3–4 and 1 mm, respectively (34). A suboptimal PTV treatment consecutive to the setup uncertainties was observed in the wedge and simple IMRT techniques. The full IMRT, performed without glancing open fields, was the technique most affected by the breast remodeling, leading to a PTV underdosage. During the whole treatment of four patients referred for helical TomoTherapy^®^, Goddu et al. reported that the uncorrected discrepancies could lead to significant dose reductions in PTV (35). The left lung exposure was not significantly modified by these setup variations. In a series of 10 patients treated with standard IMRT, the individual systematic errors were estimated at 5.7 mm along the lateral axis, 2.8 mm considering the anteroposterior plane, and 2.3 mm longitudinally (36). The individual random errors were 3.9 mm laterally, 3.5 mm vertically, and 3.2 mm along the longitudinal axis. Considering the daily PTV variations, the mean homogeneity index was 0.93.

Different techniques were assessed to reduce the uncertainties related to the patient movements: Alpha Cradle^®^ immobilization device (6), prone positioning (12, 37). Fein et al. evaluated the Alpha Cradle^®^ device in 13 patients treated with adjuvant breast radiotherapy (6). Patient setup was performed using on-line PI (6). The random and systematic errors were 4.4 and 3.9 mm for the CLD; 6.3 and 6.1 mm for the ICM, respectively. The prone and supine positions were prospectively compared in terms of setup errors and respiratory motion (12). According to Mitchell et al., in patients treated in the prone position, the use of fiducial markers put on the CT scan simulation tattoos seemed to reduce the random and systematic errors, respectively, estimated at 1.32 and 0.47 cm in the anteroposterior plan (12). Kirby et al. reported a more important systematic error in patients treated in the prone position (3.1–4.3 versus 1.3–1.9 mm in the supine position). On the other side, the chest wall and tumor bed clip motion significantly decreased (0.5 ± 0.2 mm in the prone position versus 2.7 ± 0.5 mm in the patients treated supine, *p* < 0.001) (37).

In terms of OAR exposure, the CLD is reliable to assess the ipsilateral lung irradiation. Kong et al. reported a linear correlation between the CLD and the percentage volumes of ipsilateral lung receiving 20, 30, and 40 Gy (10). The ipsilateral lung volume exposed to high doses of irradiation significantly increased when the CLD exceeded 30 mm (10). Bornstein et al. showed a statistically significant relationship between the CLD calculated on the simulation images and the volume of ipsilateral lung within the tangential fields (38).

As a conclusion, in this series of patients treated with adjuvant radiotherapy for early-stage BC, limited interfraction variations were observed using daily PI. Differences were larger along the craniocaudal axis than in the sagittal plane. The reported variations had no significant impact on the target volume coverage or OAR exposure. The 5-mm margins used to delineate the PTV seems relevant, without jeopardizing the treatment quality. Our results have to be confirmed using daily 3D imaging in a higher number of patients.

## Author Note

Presented in part at the European Society for Radiotherapy and Oncology 2012 Congress (ESTRO 31, 09th–13th May 2012, Barcelona, Spain).

## Conflict of Interest Statement

The authors declare that the research was conducted in the absence of any commercial or financial relationships that could be construed as a potential conflict of interest.
